# C-Reactive Protein in Human Atherogenesis: Facts and Fiction

**DOI:** 10.1155/2014/561428

**Published:** 2014-04-01

**Authors:** Oliver Zimmermann, Kefei Li, Myron Zaczkiewicz, Matthias Graf, Zhongmin Liu, Jan Torzewski

**Affiliations:** ^1^Cardiovascular Center Oberallgäu-Kempten, Robert Weixler Street 50, 87439 Kempten, Germany; ^2^Sino-German Heart Centre, Shanghai East Hospital, Shanghai, China

## Abstract

The role of C-reactive protein (CRP) in atherosclerosis is controversially discussed. Whereas initial experimental studies suggested a pathogenic role for CRP in atherogenesis, more recent genetic data from Mendelian randomization trials failed to provide evidence for a causative role of CRP in cardiovascular disease. Also, experimental results from laboratories all over the world were indeed contradictory, partly because of species differences in CRP biology and partly because data were not accurately evaluated. Here we summarize the published data from experimental work with mainly human material in order to avoid confusion based on species differences in CRP biology. Experimental work needs to be reevaluated after reconsideration of some traditional rules in research: (1) in order to understand a molecule's role in disease it may be helpful to be aware of its role in physiology; (2) it is necessary to define the disease entity that experimental CRP research deals with; (3) the scientific consensus is as follows: do not try to prove your hypothesis. Specific CRP inhibition followed by use of CRP inhibitors in controlled clinical trials may be the only way to prove or disprove a causative role for CRP in cardiovascular disease.

## 1. CRP and Its Role in Physiology

C-reactive protein (CRP), the prototype human acute phase protein, is a pentameric molecule consisting of 5 identical subunits of 23 kD each [1, 2]. CRP has been first identified and described by Tillet and Francis in 1930 via its ability to bind to the C-fragment of* Streptococcus pneumoniae* [3]. In acute phase response, CRP plasma concentrations, within a few hours, can raise up to 1000-fold compared to normal [1, 2]. Because of its role as the prototype acute phase protein, CRP is one of the most frequently quantified molecules in clinical medicine. It is widely used by clinicians to monitor acute phase, for example, in pneumonia, sepsis, skin and soft tissue infections, and trauma and also in controlling the patient's response to antibiotic therapy. Furthermore, CRP is an indicator of activity in autoimmune diseases [4].

Paradoxically, in spite of this wide-spread clinical use relatively little is known about the molecule's biological functions. It is interesting to note that even in well-recognized and careful reviews summarizing the current evidence on CRP and cardiovascular disease, the basic science which has been accumulated over more than 80 years and has unraveled very few major functions of CRP in the human immune system seems to be almost forgotten [5, 6].

C-reactive protein is synthesized mainly in the liver in response to interleukin-6 (and interleukin-1) [1, 2]. The two prominent CRP functions are (1) activation of the classical complement pathway via C1q binding [7]. Each 23 kD subunit contains a Ca^2+^-dependent phosphorylcholine binding site as well as a complement C1q binding site [8]. Phosphorylcholine binding induces a conformational change on the opposite site of the molecule with consecutive C1q binding and activation of the classical complement pathway (reviewed in [9]); (2) binding to human immunoglobulin Fc*γ* receptors and, hereby, opsonization of biological particles for macrophages [10–14]. Notably, these functions are also antibody functions. Therefore, it is not unlikely that CRP has been the first antibody-like molecule in the evolution of the mammalian immune system. As CRP functions have been taken over by antibodies with time, CRP may well be an atavism in the human immune system.

CRP is highly conserved in the evolution as evidenced by the atlantic horseshoe crab “*Limulus polyphemus,*” which evolved in Paleozoic Era and expresses a CRP-like molecule in its immune system [15]. Nonetheless, there are surprising species differences in CRP biology [1, 2]. For example, CRP is not an acute phase reactant in the most broadly available experimental animal model, that is, the mouse [1, 2, 16, 17]. Any attempt to overcome this problem by overexpressing human CRP in mice [17] finally causes expression of a foreign antigen in these organisms with unforeseeable consequences for the animal's immune system [17]. The latter holds true for any other animal species and also for injection of human CRP into such animals. Further problems are known for the rat model with very high basic CRP plasma levels in these animals [18]. The only smaller animal model that seems to be useful for studying CRP biology and its role in cardiovascular disease is the rabbit model. Here, CRP is an acute phase reactant and activates complement [19, 20]. With regard to this animal model three major points have to be taken into consideration. First, the cholesterol hypothesis in atherosclerosis originates from the Anichkov rabbit model [21]; secondly, cholesterol feeding induces very high CRP levels in the plasma as well as CRP deposition in atherosclerotic plaques in the rabbit model [19]; and thirdly, complement C6 deficiency in rabbits protects these animals from lesion development [22, 23]. Here, an almost ideal animal model [19, 24] (with no need to overexpress human CRP in these animals [25]) seems to exist, although the latter has not yet been fully recognized [26]. However, interesting new data from this issue presented by Szalai and colleagues tell us that it may nonetheless be wrong to dismiss any animal model from the cardiovascular disease portfolio.

## 2. CRP and Its Role in Pathology

In contrast to the large number of epidemiological studies that correlate plasma levels of high sensitivity CRP (hsCRP: CRP measured by highly sensitive assays) with various diseases, for example acute coronary syndrome, heart failure, stroke, chronic obstructive pulmonary disease, peripheral artery disease, hemodialysis, cancer, hypertension, atrial fibrillation or coronary heart disease (F. Strang, and H. Schunkert, “C-reactive-protein and coronary heart disease: All said—isn't it?,” Mediators of Inflammation, 2014), there is much less information about its molecular interactions within the affected tissues. Again, we would like to focus mainly on human material and again, the two prominent biological CRP functions, that is, complement activation and binding to human immunoglobulin Fcgamma receptors (Table 1), will primarily be discussed.

### 2.1. Atherosclerosis

Based on the identification of CRP as a cardiovascular risk marker in humans, the vast amount of experimental research with human material has been performed on atherosclerosis as the underlying cause for many cardiovascular disease entities [27–42].

*(A) Histopathology of Human Atherosclerotic Lesions*. Valid histopathology with its simple phenomenological approach may be the observational basis for any hypothesis on atherosclerotic lesion development. Although initially denied [27], CRP indeed deposits in all stages of human atherogenesis [28–31]. Notably, CRP in the lesion colocalizes with activated complement fragments [29, 31], and notably, with regard to cell types, CRP colocalizes almost exclusively with macrophages [30]. The conclusions to draw from these histopathological findings are as follows. First, CRP may be an important complement activating molecule in human atherogenesis and may thereby sustain a chronic autotoxic mechanism operating in the diseased arterial wall; second, macrophages (which strongly express Fc*γ* receptors [32]) are likely the target cells for CRP action in the arterial wall.

*(B) In Vitro Experiments*. Many* in vitro* studies have proposed diverse CRP effects on vascular cells. In these studies, some of the above mentioned rules have not been taken into account. The known biological CRP functions were not the underlying basis for the* in vitro* experiments. Consequently, CRP effects on smooth muscle cells and endothelial cells have been reported that were caused by contaminants of the applied CRP preparations, rather than by the CRP itself [33, 34]. Such publications have, unnecessarily, shed a dark light on the research topic in general [35, 36].

*(C) CRP-Mediated Opsonization of LDL for Macrophages via Fc γ* *Receptors*. Compelling evidence from* in vitro* studies on CRP-mediated opsonization of biological particles reports that (1) CRP binds to various unmodified and modified forms of LDL [37–39], (2) CRP binds to and signals via Fc*γ* receptors [11–14], and (3) LDL-bound CRP is taken up by macrophages via Fc*γ*R dependent and Fc*γ*R independent pathways [40].

In brief, CRP colocalization with macrophages in the lesion, high expression levels of Fc*γ*Rs on macrophages, and CRP-mediated LDL uptake into macrophages suggest that CRP opsonizes LDL for macrophages. Thereby, CRP may be deeply involved in foam cell formation in atherogenesis [40].

*(D) CRP Mediates Complement Activation in Atherogenesis*. Compelling evidence from* in vitro* studies on CRP and complement activation in atherogenesis suggests that (1) CRP conformation, either pentameric or monomeric, regulates complement activation in the vessel wall [41] and (2) CRP may also have protective effects by haltering modified lipoprotein-mediated complement activation before detrimental terminal sequence [42].

In brief, colocalization of CRP with activated complement fragments in atheroslerotic lesions and complement activation by CRP/LDL complexes* in vitro* strongly suggest that lipoprotein-bound CRP is intimately involved in complement activation in atherogenesis.

*(E) Genetic Studies on CRP and Cardiovascular Disease*. Concerning genetic studies on CRP and cardiovascular disease we refer to another article in this issue (Strang et al.). It is, however, important to note that results from Mendelian randomization trials do not support the concept that CRP is causally involved in atherogenesis and its sequelae [43–45]. Still, the limitations of Mendelian randomization need to be taken into account [46, 47], especially in case of a molecule like CRP whose synthesis in the liver is very complexly regulated on the transcriptional level [48–50].

### 2.2. CRP and Nonvascular Disease

#### 2.2.1. Cardiology

A contribution of CRP to pathogenesis has been suggested for two other cardiovascular disease entities, that is, myocardial infarction and dilated cardiomyopathy.

*(A) Myocardial Infarction*. CRP plasma levels significantly rise after myocardial infarction indicating the human body's acute phase response [51, 52]. CRP deposits in human myocardial scars following coronary occlusion [51, 52]. In this location, CRP again colocalizes with activated complement fragments suggesting that CRP-mediated complement activation in necrotic tissue is a more general phenomenon. While in rats CRP-mediated complement activation seems to contribute to myocardial damage and while inhibition of CRP by a CRP-cross-linker may be protective [53], complement inhibition in human myocardial infarction has never been conclusively demonstrated to be beneficial [54]. The therapeutical focus in myocardial infarction should certainly be laid on reopening of the clotted coronary artery.

*(B) Dilated Cardiomyopathy*. Presence and distribution pattern of myocardial CRP in patients suffering from nonischemic chronic cardiomyopathy have been investigated [55]. Myocardial biopsies from dilated cardiomyopathy (DCM) patients either with or without accompanying chronic myocardial inflammation or virus were immunohistochemically studied for CRP and C5b-9 [55]. Myocardial CRP was detected in almost one-third of the patients. CRP again colocalized with macrophages and the terminal complement complex C5b-9. As there was no correlation with hsCRP plasma levels and as spacial distribution of myocardial C5b-9 was much broader than CRP distribution, CRP may not be the only myocardial complement activator in DCM.

#### 2.2.2. Neurology

CRP and complement proteins were also detected in cerebral lesions in Alzheimer's disease [56]. Compared with normal brains, CRP mRNA levels were elevated in brains of Alzheimer patients, and thus it was concluded that CRP might be produced within the brain rather than being derived from the plasma [57]. CRP, again, seems to activate the complement system in brains and may lead to chronic neuroinflammation which then may cause neuronal death in Alzheimer's disease. CRP was also detected in brain lesions of patients who died of acute ischemic stroke or spontaneous intracerebral hemorrhage [58]. This observation was associated with a significant increase of hsCRP plasma level indicating the organism's acute phase response.

#### 2.2.3. Rheumatology

CRP was also detected in synovial biopsies from patients with rheumatoid arthritis [59]. The nuclei of synoviocytes and histiocytes in the rheumatoid synovial membrane were found to bind CRP. Synovial-bound CRP was not of local origin and colocalized with antibodies and complement C3. Nonarthritis patients did not have CRP within their synovial membranes. Again this observation may indicate an active or causative role for CRP in rheumatoid arthritis [59]. Other studies showed CRP deposition in the synovial fluid of different joint diseases [60, 61]. There is evidence that CRP is either being selectively bound in synovium or specifically consumed in synovial fluid. Thus, CPR may play an important role also in the inflammatory process of this disease [61].

## 3. Targeting C-Reactive Protein for the Treatment of Cardiovascular Disease

In spite of huge effort to develop CRP inhibitors, up to the present day no laboratory or pharmaceutical company worldwide has succeeded in developing a specific anti-CRP agent that is readily applicable in humans. As a consequence, less specific anti-inflammatory drugs like IL-1*β* antibodies or methotrexate are currently tested in clinical trials to prevent patients from the progression of cardiovascular disease [62, 63]. In our view, these trials will very unlikely be successful because the side effects of these approaches may eliminate the potential therapeutic benefits. The trials, however, are ongoing and therefore we do not know what the outcome of the studies will be.

Assumed that CRP is an atavism in the human immune system (see above), specific CRP inhibition may be less immunosuppressive and thus the only way to proceed. Specific inhibition, however, is demanding and will probably depend on future development of novel pharmaceutical strategies to target biomolecules. Principle strategies include (1) cross-linking of CRP subunits, (2) antisense strategies, (3) blockage of CRP-mediated complement activation, (4) blockage of CRP receptors, and (5) inhibition of CRP synthesis.

*Cross-linking of CRP subunits* has been attempted in the past [53]. Although originally touted as a major breakthrough, the molecule that resulted from these attempts is a more or less ubiquitous cross-linker and has never found its way into human application. Use of* antisense strategies* obtained promising results in two animal models and antisense molecules have also successfully lowered CRP plasma levels in healthy volunteers [64], [1, in this issue]. Although one of the challenges of this approach is how to apply such molecules continuously over years, the approach seems promising.* Inhibition of CRP-mediated complement activation* by competitive blockage of the C1q binding site has been tried but was not successful due to steric reasons. In view of the identification of Fc*γ* receptors as CRP receptors and the expectable side effects,* blockage of CRP receptors* seems not reasonable. Finally, high throughput screening (HTS) with a hepatoma cell line stably transfected with the CRP promoter surprisingly resulted in the identification of cardiac glycosides as potent* transcriptional CRP inhibitors *[65]. This* in vitro* result is difficult to interpret. Whether the* in vitro* effect also applies* in vivo* in humans is currently under investigation. The latter can easily be done and is ethically justified in heart failure patients, because cardiac glycosides have been used in cardiac insufficiency for 230 years [66] and, according to heart failure guidelines, still provide an additive treatment option in NYHA classes III and IV [67].

Since generation of induced pluripotent stem (IPS) cells has recently facilitated HTS with primary human cells [68] it may be worthwhile to repeat HTS for CRP synthesis inhibitors with hepatocytes derived from IPS cells.

In summary it can be stated that, as CRP is also involved in the pathogenesis of other diseases (see above), the molecule may be a rewarding drug target. Future technologies in drug development may facilitate achievement of this demanding goal.

## Figures and Tables

**Table 1:** Colocalization of CRP, complement components, and Fc*γ*R/macrophages as a general feature in histopathology of human disease.

Disease	CRP	Complement components	Fc*γ*R/macrophages
Atherosclerosis	+	+	+
Myocardial infarction	+	+	+
Dilated cardiomyopathy	+	+	+
Alzheimer's disease	+	+	+
Ischemic stroke	+	?	?
Rheumatology	+	+	+
